# Multi-Objective Location and Mapping Based on Deep Learning and Visual Slam

**DOI:** 10.3390/s22197576

**Published:** 2022-10-06

**Authors:** Ying Sun, Jun Hu, Juntong Yun, Ying Liu, Dongxu Bai, Xin Liu, Guojun Zhao, Guozhang Jiang, Jianyi Kong, Baojia Chen

**Affiliations:** 1 Key Laboratory of Metallurgical Equipment and Control Technology, Ministry of Education, Wuhan University of Science and Technology, Wuhan 430081, China; 2 Research Center for Biomimetic Robot and Intelligent Measurement and Control, Wuhan University of Science and Technology, Wuhan 430081, China; 3 Hubei Key Laboratory of Mechanical Transmission and Manufacturing Engineering, Wuhan University of Science and Technology, Wuhan 430081, China; 4 Hubei Key Laboratory of Hydroelectric Machinery Design and Maintenance, China Three Gorges University, Yichang 443002, China

**Keywords:** deep learning, target tracking, visual SLAM, multi-objective location, semantic mapping

## Abstract

Simultaneous localization and mapping (SLAM) technology can be used to locate and build maps in unknown environments, but the constructed maps often suffer from poor readability and interactivity, and the primary and secondary information in the map cannot be accurately grasped. For intelligent robots to interact in meaningful ways with their environment, they must understand both the geometric and semantic properties of the scene surrounding them. Our proposed method can not only reduce the absolute positional errors (APE) and improve the positioning performance of the system but also construct the object-oriented dense semantic point cloud map and output point cloud model of each object to reconstruct each object in the indoor scene. In fact, eight categories of objects are used for detection and semantic mapping using coco weights in our experiments, and most objects in the actual scene can be reconstructed in theory. Experiments show that the number of points in the point cloud is significantly reduced. The average positioning error of the eight categories of objects in Technical University of Munich (TUM) datasets is very small. The absolute positional error of the camera is also reduced with the introduction of semantic constraints, and the positioning performance of the system is improved. At the same time, our algorithm can segment the point cloud model of objects in the environment with high accuracy.

## 1. Introduction

With the development of artificial intelligence and neural network, the status of simultaneous localization and mapping technology has been growing, and now, it has become a research hotspot in the field of computer vision and robotics, which has made remarkable achievements in the scientific and academic fields [1,2,3,4]. SLAM has been applied in many aspects of our lives, such as medical services, virtual/augmented reality, drones, autonomous driving, etc. [5,6]. Due to the rapid development of computer vision and deep learning, the use of semantic information in SLAM, which is called semantic SLAM, has become a very promising and challenging topic [7,8,9,10,11,12,13]. Semantics and SLAM seem to be two independent modules, but they are not. They influence and help each other in many ways [14,15,16]. On the one hand, the mapping and positioning of the classical SLAM system are mostly based on the geometric matching at the pixel level, lacking a priori information to help the sensor locate the objects [17,18]. Data association can be improved from the traditional pixel level to the object level with the help of semantic information, which can help improve the positioning accuracy in complex scenes [19,20,21]. Compared with the traditional physical geometric information, semantic information has consistency and stability and is less affected by environmental transformations. Additionally, it is suitable for image matching in both static and dynamic environments. On the other hand, the semantic information obtained by the semantic segmentation algorithm for the same object may be different at different times and from different angles, especially for objects with similar shapes. The SLAM system can provide constraints for the extraction of semantic information to improve the accuracy of semantic understanding [22]. In general, it is computationally cheaper to accomplish data association and camera pose estimation through objects than through feature points, as there are many fewer objects compared to the number of 3D points in a map.

Existing SLAM systems can already construct traditional two-dimensional (2D) maps of the environment, and many are capable of building 3D sparse point cloud maps and dense point cloud maps of the scene. With these maps, robots can carry out low-level tasks, such as localization and navigation [23,24]. However, sophisticated interactions between a robot and the surroundings require three-dimensional (3D) semantic information about the environments. The image processing methods based on deep learning, including target detection, semantic segmentation and instance segmentation, are important and promising steps to address this problem. Combined with semantic information, the SLAM system can help mobile robotics locate itself more precisely and significantly improve the ability to interact with the environment, which means that a task like “bring me an apple on the table” can be performed effectively [25]. The concept of semantic maps was first introduced at the beginning of the 21st century [26]. Most of the current semantic maps built by semantic SLAM are scenario-oriented semantic maps. The scenario-oriented semantic maps belong to pixel-level 3D semantic maps where each map point carries semantic information, making it easier for robots to understand the scene, such as background, wall, table, door and so on [27,28,29]. This way of building maps can construct a more expressive map of the environment to assist the robot to better understand the environment as a whole, but it is not good for the robot to recognize the individuals in the environment. Robots cannot interact with individuals in the environment because they cannot distinguish between different individuals in the environment, which limits the application of intelligent robots to a certain extent [30,31,32]. The main contributions of this paper are as follows:

(1) Propose a method of target tracking combined with instance segmentation, which can segment the objects in an image and track each object to establish data associations for different frames.
(2) Propose a method for constructing objected-oriented semantic maps.
(3) Propose a method for reconstructing multiple objects in 3D space.
(4) Propose a method for optimizing the camera pose using object constraints.

In the rest of the paper, the second part is related works, the third part is the method and theory, the fourth part is the experiment and analysis, and the last part is summary and expectations.

## 2. Related Work

### 2.1. Image Segmentation Networks

Semantic segmentation goes further on the basis of target detection and needs to distinguish each pixel point in the image to segment and distinguish different classes of objects. Instance segmentation pursues more precise segmentation based on semantic segmentation to distinguish each object of the same category. Starting from the proposal of fully convolutional networks (FCN) in 2015, the classical neural-network-based image segmentation algorithms start to appear. Long et al. propose FCN, which replaces the full convolutional layer with the full connection layer in the convolutional neural network (CNN) to build an end-to-end, pixel-to-pixel semantic segmentation network [33]. Wang et al. propose RDS Net, which introduces three modules, including the target frame auxiliary instance mask relationship module, the mask pruning module and the mask refinement target positioning module, and it designs a dual flow network to overcome the low resolution of the instance mask to a great extent [34]. Cai et al. propose Cascade R-CNN, which adds a split branch for each cascade stage, extending the cascade architecture to instance segmentation tasks [35]. Jiang et al. introduce two branches on the basis of Mask R-CNN [36] and propose a post-processing refinement module based on the overall nested edge detection best possible recall (BPR) to improve the boundary quality of Mask R-CNN [37]. Instance segmentation tasks are also widely used in remote-sensing images, face detection and other scenes.

### 2.2. Visual SLAM

The research on visual SLAM includes the positioning of the camera and the construction of an environmental map. Kerl et al. propose DVO-SLAM, a dense visual SLAM method for RGB-D cameras that minimizes both the photometric and the depth error over all pixels [38]. Endres et al. propose RGBD-SLAM v2, which calculates the camera motion between two frames by feature matching and iterative closest point (ICP) methods in the front end and performs pose graph optimization with loop detection in the back end, and it can build 3D maps with high accuracy [39]. McCormac et al. propose Fusion++, which is built from modules for image-based instance segmentation, truncated signed distance function (TSDF) fusion and tracking and pose graph optimization, and create a long-term map, which focuses on the most important object elements of a scene [40]. Mur-Artal et al. propose ORB-SLAM2, which is the excellent successor of parallel tracking and mapping (PTAM) and achieves the first parallelization of three threads for real-time tracking, local bundle adjustment (BA) optimization and global loop detection, and it uses the Oriented FAST and Rotated BRIEF (ORB) features for feature point extraction, matching, loop detection and relocalization [41]. Qin et al. propose the monocular visual-inertial SLAM system called Vins-Mono, which belongs to the optimization-based tight-coupling method [42]. Whelan et al. propose Elasticfusion, which adopts the mapping method of continuously optimizing reconstruction to improve the accuracy of reconstruction, and the map is represented by the surfel model [43]. Dou et al. propose Fusion4d, which uses several depth cameras to reconstruct rapidly changing non-rigid bodies [44]. Muhammet et al. adopt visual-inertial odometry (VIO), a low-cost sensor fusion method that integrates inertial data with images using a deep-learning (DL) framework to predict the position of an unmanned aerial system (UAS) [45]. Cao et al. propose GVINS, which tightly integrate GNSS raw measurements with visual and inertial information to achieve real-time and drift-free state estimation [46]. In general, the research direction of SLAM is more focused on improving the positioning performance of the system.

### 2.3. Semantic Map

Some scholars proposed the concept of semantic maps at the beginning of the 21st century and elaborated that building environment maps with semantic information could enhance the perception and interaction capabilities of robots. In recent years, more and more researchers have combined deep-learning methods with SLAM technology to extract semantic information of the environment using target detection, semantic segmentation and other algorithms. Then, they are integrated into the environment map to construct a semantic map of the environment. Semantic maps can be broadly divided into scenario-oriented semantic maps and object-oriented semantic maps.

The scenario-oriented semantic maps divide the environment into several semantic parts in a holistic way. Li et al. combine the DeepLab network [47] with LSD-SLAM [48] to build semi-dense semantic maps through pixel-level semantic segmentation without any prior depth information. The semantic annotations are transferred into the 3D map and regularized with a CRF process. However, the initialization of the system still needs to be optimized [49]. McCorma et al. propose a method called SemanticFusion to construct pixel-wise semantic maps in indoor scenes. This method optimizes the semantic segmentation results of a single image based on the matching relationship of image feature points output by the SLAM algorithm, but the improvement is very limited and requires great computational resources [50]. Brucker et al. propose a method to determine the room type based on information such as the type and distribution of objects within the environment and propose a method to calculate the degree of association between objects and rooms, which helps improve the execution efficiency of robot interaction tasks [51]. Chen et al. propose a probabilistic model to consider the semantic information of all frames to estimate the semantics of each map point. In addition, the authors use temporal motion information to argue whether a map point is dynamic or static [52]. Zhao et al. propose a pixel–voxel network to obtain global context information by using PixelNet to exploit the RGB image and, meanwhile, preserve accurate local shape information by using VoxelNet to exploit the corresponding 3D point cloud. Subsequently, each point on the 3D map is identified and semantically tagged [53]. CNN-SLAM naturally blends the dense depth map predicted by CNN with the depth measurements obtained by direct monocular SLAM to obtain an improved depth map, which is used to build a scene-oriented semantic map [54].

Object-oriented semantic maps are composed of individual instances, which allows the robot to manipulate and maintain each object in the map. Salas-Moreno et al. propose SLAM++ to create a map with semantically defined objects, but it relies on a predefined database and hand-crafted template models [55]. Hoang et al. propose Object-RPE to solve the problem of constructing semantic maps when there are occlusions and environmental clutter in the environment, and it has better robustness [56]. Sunderhauf et al. propose an object-oriented semantic mapping method based on the combination of SSD [57] and ORB-SLAM2, which maintains and updates the point cloud for each class of objects during the runtime. However, it is not possible to maintain and update each object in the map individually, which makes it difficult for the robot to distinguish between objects of the same category in practical applications [58]. Grinvald et al. propose an online volume-instance-aware semantic map building system based on RGB-D data, where the exact segmentation of objects is obtained by joint inference of geometric and semantic cues, and finally, the global object-level semantic map is obtained by fusing local information. However, the pose estimation errors accumulate during the operation of the system, leading to an increased impact on the map [59]. Li et al. directly use the mapping relationship between voxels and pixels to obtain the semantic categories of voxels, thus reducing the computational effort in semantic fusion and improving the real-time performance of the algorithm to a certain extent, but the accuracy of the semantic map is slightly poor [60]. Liu et al. propose to combine YOLO V4 [61] with RGB-DSLAM to perform dense mapping and object location, but the boundaries of the constructed map are not perfect [62]. Runz et al. propose Mask-Fusion, which is a method of semantic mapping based on instance segmentation in dynamic scenes. This method combines instance segmentation and geometric segmentation to solve the problem of rough edges, but the segmentation method used requires a large amount of computational resources [63]. Dorian et al. propose a monocular SLAM system for perceptible objects, which uses the binary bag of words (a database containing 500 3D object models) to complete target recognition and construct 3D semantic maps, but the system causes obvious errors in the maps when the initialization fails [64].

## 3. Method

Our method is divided into two main parts: semantic information extraction and semantic mapping. In the section of semantic information extraction, the classical target tracking algorithm Deepsort [65] and the instance segmentation algorithm Mask R-CNN [36] are combined to extract the 2D semantic information of the target object and establish data associations between the images, which provide a priori information for subsequent camera pose optimization and point cloud fusion. In the section of semantic mapping, the RGB-D version of ORB-SLAM2 [41], an open-source SLAM system, is used for tracking and mapping, which uses both RGB and depth information for sparse tracking. Semantic information is added to the newly created semantic mapping thread of ORB-SLAM2 to build object-oriented semantic maps, reconstruct the target objects in the maps and optimize the camera pose, which is critical and applicable in the field of human–computer interaction. We are cautious about terming our method “semantic SLAM”, as its usual task consists of two aspects: SLAM helping semantics and semantics helping SLAM. Our method contains one of the two directions—semantics helping SLAM—including localization and mapping.

### 3.1. Target Tracking Based on Instance Segmentation

**Figure 1:**
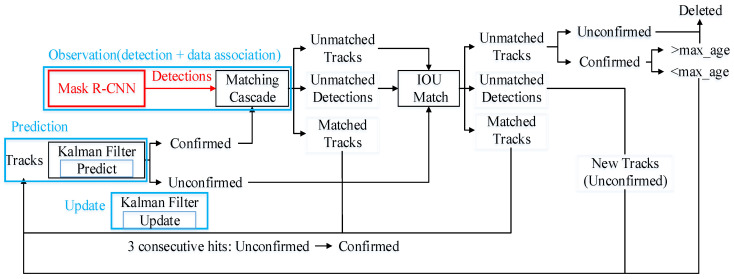
The frame diagram of Deepsort with Mask R-CNN.

Mask R-CNN [36], an instance segmentation algorithm, is used to obtain 2D semantic information in RGB images in our method, which is used as the observation data for the target tracking algorithm to track the target objects to establish the data association in a video sequence. The extracted 2D semantic information includes semantic label, tracked identification (ID) and 2D pixel coordinates, where the 2D pixel coordinate information of the objects will be recorded in txt files in a hierarchical manner according to the timestamp of the image, the semantic classification and tracking ID of the object. These pixel coordinates are extracted from the object’s mask, and the two coordinates of a point occupy one line of the txt file. The frame diagram of Deepsort with Mask R-CNN is shown in Figure 1.

Deepsort [65], a classical multi-objective tracking algorithm, is mainly used to track and predict vehicles and pedestrians in combination with target detection. The core processes of Deepsort include prediction, observation and update. The prediction thread performs prediction for the next frame to obtain the prediction box of the target objects. The observation thread performs target detection for the current frame to obtain the detection box of the target objects and establish data association with the objects in the previous frame. There is some error between the results generated by the above two threads and the real results. The update thread combines the results of the prediction thread and the observation thread to update the results, making the final error smaller than that of the single thread. The prediction thread and update thread of Deepsort can predict the position of tracks at the next time and update the predicted location based on detections by using Kalman filtering. Kalman filtering uses a linear homogeneous model, which means the movement of the frame, the change in the size and deformation of the frame are considered to change linearly and homogeneously. In Deepsort, the tasks of the prediction thread can be expressed as the following formula.

(1) $${\bar{\hat{x}}}_{k}=A{\hat{x}}_{k-1}$$

(2) $${\bar{P}}_{k}=AP_{k-1}A^{T}+Q$$

where Formula (1) is used to compute the mean vector ${\bar{\hat{x}}}_{k}$ of the prediction box at frame k; Formula (2) is used to compute the covariance matrix ${\bar{P}}_{k}$ of the prediction box at frame k; A represents the motion matrix and is used to predict the state of the system at the next time. The tasks of the update thread include computing the Kalman gain, updating the estimate via measurement and updating the error covariance, which can be expressed as the following formula, respectively.

(3) $$y=z_{k}-H{\bar{\hat{x}}}_{k}$$

(4) $$S=H{\bar{P}}_{k}H^{T}+R$$

(5) $$K_{k}={\bar{P}}_{k}H^{T}S^{-1}$$

(6) $${{\hat{x}}^{\prime }}_{k}={\hat{x}}_{k}+K_{k}y$$

(7) $$P_{k}=(I-K_{k}H){\bar{P}}_{k}$$

where $z_{k}=[m,n,l,h]$ represents the mean vector of the detection box at frame k; the four parameters represent the horizontal and vertical coordinates of the upper left corner, length and width of the detection box; y represents innovation; H represents the measurement matrix; in Formula (4), R represents the noise matrix of the detector; in Formula (5), $K_{k}$ represents the Kalman gain at frame k and is used to estimate the importance of the error; Formulas (6) and (7) are used to compute the updated mean vector and the covariance matrix. The observation thread includes the results detected by Mask R-CNN and data association. The results detected by Mask R-CNN, which include the box, mask, class and score of each object in the picture, are regarded as the observed data of Kalman filtering to match one by one with the prediction box in the previous frame. The match algorithm includes the intersection-over-union (IOU) match and the cascade match, and the cost function includes the Mahalanobis distance and cosine distance. If both distances are as small as possible, and the boxes and features are similar, it is considered that the two boxes are the same thing. The successful pairing of the detection box and the prediction box means that the previous frame and the next frame are successfully tracked. Whether the objects can be tracked successfully is greatly influenced by the detection results.

### 3.2. Improved Dense Mapping Based on ORB-SLAM2

ORB-SLAM2 [41], one of the very classic and practical systems in modern SLAM work, which represents a peak of the mainstream feature point SLAM, is used to construct semantic maps in our method. The whole system is computed around ORB features, with fast computation speed, rotational invariance and scale invariance. We propose to add a new point cloud mapping thread in ORB-SLAM2 to upgrade the original sparse map to a dense semantic map. The prerequisite for mapping is to calculate the camera’s pose. Therefore, the camera pose estimation problem needs to be solved first. In SLAM, the pose of the camera can be initially estimated in the visual odometry, which may have some errors. In order to optimize the camera pose x, SLAM is transformed into an optimization problem. The equation of observation and motion in visual SLAM can be expressed as

(8) $$\left\{ \begin{array}{l} x_{i}=f(x_{i-1},u_{i})+\omega_{i}\\ z_{i,j}=h(y_{j},x_{i})+v_{i,j} \end{array}\right.\;\;\;\;i=1,2,\ldots \ldots ,N,\;j=1,2,\ldots \ldots ,M$$

where $x_{i}$ represents the camera pose at frame i; $y_{j}$ represents the jth road sign; $z_{i,j}$ represents the data generated by the jth road sign observed at frame i; $u_{k}$ represents the motion data at frame i; $w_{i}$ and $v_{i,j}$ represent the motion noise and observation noise generated at frame i. If there are no motion data, the camera pose can also be optimized by observing the data. Considered in terms of least squares, then, the error of the observation can be expressed as

(9) $$e_{ij}=z_{i,j}-h(\xi_{i},y_{j})$$

After considering the observed data at other moments, the overall cost function of the observed error to be optimized can be expressed as

(10) $$\frac{1}{2}\sum_{i=1}^{m}\sum_{j=1}^{n}\left|\right|e_{ij}|{|}^{2}=\frac{1}{2}\sum_{i=1}^{m}\sum_{j=1}^{n}\left|\right|z_{ij}-h(\xi_{i},y_{j})|{|}^{2}$$

where $z_{i,j}$ represents the observed data of the jth road sign $y_{j}$ at frame i; $\xi_{i}$ represents the Lie algebra corresponding to the camera pose at frame i. $e_{ij}$ represents the observation error arising from the observation of road sign j at frame i. m denotes the number of images involved in the calculation. n denotes the number of road signs involved in the calculation. Solving this least-squares calculation is equivalent to making adjustments to both the camera pose ξ and the road signs y, which is also known as BA. The point cloud mapping thread converts the coordinates of all pixels with a depth value greater than 0.01 on the key frame to 3D coordinates using the information of the corresponding depth value, camera pose and camera internal parameter matrix. The transformation can be divided into four steps:

(1) World coordinates to camera coordinates

(11) $$\vec{P_{C}}=\left[\begin{array}{l} X_{C}\\ Y_{C}\\ Z_{C} \end{array}\right]=\left[R\;\;\;t\right]\left[\begin{array}{l} X_{W}\\ Y_{W}\\ Z_{W}\\ 1 \end{array}\right]$$

(2) Camera coordinates to the normalized coordinates *P_C_*

(12) $$P_{C}={\left[X_{C}/Z_{C},Y_{C}/Z_{C},1\right]}^{T}$$

(3) The points in the normalized plane are distorted, and the distortion parameter is

(13) $$D=(k_{1},k_{2},k_{3},p_{1},p_{2})$$

(4) Normalized coordinates to pixel coordinates

(14) $$P_{uv}=\left[\begin{array}{l} u\\ v\\ 1 \end{array}\right]=\left[\begin{array}{cccc} f_{x} & 0 & u_{0} & 0\\ 0 & f_{y} & v_{0} & 0\\ 0 & 0 & 1 & 0 \end{array}\right]P_{C}$$

The total conversion formula is

(15) $$\left[\begin{array}{l} u\\ v\\ 1 \end{array}\right]=\frac{1}{Z_{C}}\left[\begin{array}{cccc} f_{x} & 0 & u_{0} & 0\\ 0 & f_{y} & v_{0} & 0\\ 0 & 0 & 1 & 0 \end{array}\right]\left[\begin{array}{cc} R & t\\ \vec{0} & 1 \end{array}\right]\left[\begin{array}{l} X_{W}\\ Y_{\;W}\\ Z_{W}\\ \;1 \end{array}\right]$$

where $f_{x},f_{y},u_{0},v_{0}$ represent the internal parameters of the camera; R represents the rotation matrix; t represents the translation matrix; $Z_{C}$ represents the depth value; (u,v) represent the pixel coordinates; $\left(X_{W},Y_{W},Z_{W}\right)$ represent the corresponding world coordinates. $\left(X_{C},Y_{C},Z_{C}\right)$ represent the corresponding camera coordinates. The point cloud mapping thread can generate a dense point cloud of the environment using the key frame according to the transformation relationship between the world coordinate system, the camera coordinate system and the pixel coordinate system. The key frame is representative of a series of local ordinary frames and is responsible for recording local information. The mapping method based on the key frame can retain most of the mapping information, which compensates for the shortage of carrying too little information in the sparse map, and it can also avoid the problems of large amount of calculation and information redundancy caused by calculating each image.

### 3.3. Semantic Mapping Based on Semantic SLAM

**Figure 2:**
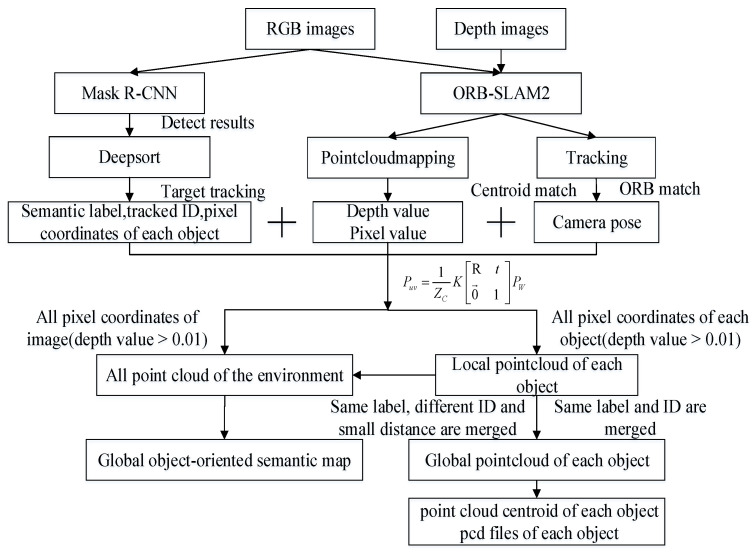
The frame diagram of the object-oriented semantic mapping system.

The contents of Section 3.1 and Section 3.2 can be combined to build object-oriented semantic maps. The frame diagram of the object-oriented semantic mapping system is shown in Figure 2.

The specific steps of the system can be divided into three steps. The first step is the extraction of semantic information carried out by Mask R-CNN and Deepsort. The input RGB images are detected and segmented by Mask R-CNN, and the detected results are put into the Deepsort as observation data, including the box, class, mask and score of each object. Deepsort tracks each target and assigns an ID to each tracking target. The 2D pixel coordinates of each object on each image are recorded in txt files. These txt files contain information such as the timestamp, class names, tracked ID and the pixel coordinates corresponding to each ID and are put into ORB-SLAM2.

The second step is the construction of a semantic map using the extracted semantic information and ORB-SLAM2. When the ORB-SLAM2 system receives a key frame, the pixel coordinates in the corresponding txt files are converted by the point cloud mapping thread to 3D coordinates with semantic colors in the camera coordinate system, and the other pixel coordinates in the key frame are transformed into 3D coordinates with the original color. The map obtained in this way is a local semantic map that can be converted to a point cloud map in the world coordinate system based on the current camera pose. In order not to affect the running speed of SLAM, the RGB images, depth maps, local semantic maps and camera poses corresponding to key frames are stored in separate containers, and there is a separate calculation process of traversing information and building maps. After filtering, all local maps are transformed into point cloud maps in the world coordinate system according to the transformation matrix. Then, these maps are fused into a global semantic map.

The third step is the reconstruction of the target object. In order to operate and maintain each object in the map, we need to know the location of each object. A possible method is to convert the coordinates of each object extracted in the first step into a 3D point cloud and subsequently fuse the point clouds with the same ID at different moments to construct a point cloud model of the target object. An obvious problem, however, is that objects with different IDs may belong to the same object because the ID may change during the process of target tracking. To collect point cloud information from multiple angles of an object to reconstruct the object, the point cloud of the same object with different IDs needs to be fused according to the theory of multi-view geometry. In this paper, we calculate the centroids of the point clouds and calculate the Euclidean distance between the centroids of the point clouds; then, the two point clouds are considered as the same point clouds if the distance of the two centroids is less than 0.1 m. The calculation formulas are as follows.

(16) $$x_{c},y_{c},z_{c}=\sum_{i=1}^{n}x_{i},\sum_{i=1}^{n}y_{i},\sum_{i=1}^{n}z_{i}$$

(17) $$d_{12}=\sqrt{{\left(x_{1}-x_{2}\right)}^{2}+{\left(y_{1}-y_{2}\right)}^{2}+{\left(z_{1}-z_{2}\right)}^{2}}$$

where $\left(x_{c},y_{c},z_{c}\right)$ is the centroid of the point cloud; $\left(x_{i},y_{i},z_{i}\right)$ is the 3D coordinate of the *i*th point. n represents the number of points in the point cloud.$d_{12}$ represents the Euclidean distance between two centroids.$x_{1},y_{1},z_{1}$ and $x_{2},y_{2},z_{2}$ represent the 3D coordinates of the two centroids. There will be outlier points in the actual calculation process. For example, the boundary information extracted by semantic segmentation is not perfect and contains some points that do not belong to the object, which are converted into 3D points with a large distance from the 3D points of the object and should be eliminated; otherwise, the calculated centroid is inaccurate. In this paper, the point clouds are filtered by using the filtering method of rejecting outlier points. The general idea is as follows:

(1) For each point in the point cloud, we calculate the average distance d from it to all the points in its K-neighborhood.
(2) The distances calculated above are assumed to obey a Gaussian distribution. The corresponding points with distance values outside the standard range (defined by the mean and variance of the sample) are defined as outliers and should be removed from the datasets.

The threshold for the outlier judgment is calculated as

(18) t=m+k∗s

where t is the judgement threshold, m represents the mean value of the distance sample, s represents the standard deviation of the distance sample. k represents the parameter used to adjust the size of the threshold. The smaller the k, the smaller the threshold and the more obvious the filtering effect. However, this does not mean that the smaller the k, the better the filtering effect. When k decreases, the boundary points of the objects will be filtered out, which is not the desired result. As the local maps of the objects are fused, a large number of duplicate or very close points will be added to the point cloud, which will eventually result in a very dense point cloud and take up a lot of memory space. Too dense point clouds are not meaningful for object reconstruction and need to be filtered. In this paper, the point clouds are filtered by using the voxel filtering method. The core idea is to divide the point cloud into multiple voxels (three-dimensional cubes) and use the points nearest the center of the voxel to represent all the point clouds within the voxel. This filtering method allows for a significant reduction in the number of 3D points within the point cloud while preserving the shape characteristics of the point cloud. In addition, the instance segmentation algorithm has a misdetection in the process of detection because the features of the same object from different angles are not the same. For example, in the detection process, teddy bears are sometimes detected as people, and mouses are sometimes detected as cups. To solve this problem, we consider that the object can be correctly detected when its corresponding ID appears at least 3 times, and we consider this object to be an object that needs to be reconstructed. The point clouds of the correctly detected objects are saved, and whenever the objects carrying the same ID or with a distance of less than 0.1 m from the centroids of the existing point clouds reappear, the newly transformed point clouds are added to the corresponding existing point clouds. Finally, we can obtain the point cloud models of all objects. The position of an object in a three-dimensional space is represented by the centroid of the corresponding point cloud.

### 3.4. Camera Pose Optimization Based on Semantic SLAM

In addition to using geometric features, our approach uses detected semantic objects as landmarks and tracks and matches objects in different images, which elevates the data association problem in SLAM from the traditional pixel level to the object level and provides object constraints for camera pose estimation, thus optimizing the camera pose. After obtaining the pixel coordinates of the object, the pixel coordinates are transformed into 3D coordinates under the camera coordinate system by the camera internal reference, and thus, the 3D point cloud of the object is obtained. The centroid of the object’s point cloud is used to represent the object’s position in the 3D space. In Section 3.1, we use the target tracking algorithm to establish data association between different images and assign tracking IDs to the objects, so that objects carrying the same ID in different images are considered as the same object and are used for object matching. Our approach uses the point cloud centroid of an object to represent its position in a three-dimensional space and is used for object matching. Compared with feature matching, object matching is less influenced by the environment and has better robustness. The 3D to 3D method of calculating the poses is the ICP algorithm. The goal remains to calculate the camera pose x. The camera pose can also be expressed using the Lie algebra ξ. Suppose there are a set of well-matched 3D point cloud centroids, which are as follows:

(19) $$P=\{p_{1},p_{2}\cdots ,p_{n}\},\;\;\;\;P^{\prime }=\{{p^{\prime }}_{1},{p^{\prime }}_{2}\cdots ,{p^{\prime }}_{n}\}$$

When expressing the camera pose in terms of the Lie algebra ξ, the objective function can be written as follows:

(20) $$f(\xi )=\frac{1}{2}\sum_{i=1}^{n}||(p_{i}-\exp(\xi^{\wedge })p_{i}{}^{\prime }){\left|\right|}_{2}^{2}$$

where $p_{i},p_{i}{}^{\prime }$ represent a pair of centroids matching the 3D coordinates; $\exp(\xi^{\wedge })$ represents the left multiplication perturbation model of the Lie algebra. n signifies that there are n pairs of matching points. The goal is to find the ξ that minimizes f(ξ). Object matching can provide more stable observation data. Correct centroid matching can add constraints to the pose estimation and optimize the camera pose, while incorrect centroid matching can do the opposite, so more correct centroid matching is needed for pose optimization. This paper proposes a method to calculate the probability that two point clouds are point clouds of the same object, which is used to select the correct centroid matching.

Suppose there are two sets of point clouds A, B; first, transform the two point clouds into the same coordinate system by the estimated transformation matrix of the two images and build A as an octree; then, iterate through all points in B and query whether this point exists in the corresponding voxel of A (i.e., whether the point clouds of A and B exist in the same voxel); if it does exist, the point is the overlapping point cloud of B. The above method can obtain the overlapping point clouds in A and B. The degree of overlap of the two point clouds can be expressed as the following equation.

(21) $$M=\frac{\mathrm{AB}}{A+B-\mathrm{AB}}$$

where AB represents the overlap of point cloud A and point cloud B; M represents the degree of overlap. There are several ways to evaluate whether A and B are point clouds of the same object.

(1) Both have the same ID.
(2) The distance between the two centroids in the same coordinate system is less than 0.1 m.
(3) The degree of overlap between the two, M, is greater than 0.8.

In practice, although the target tracking algorithm provides a priori information for object matching, it still has limitations. Since the IDs will switch during target tracking, objects with different IDs may belong to the same object. Likewise, objects with the same ID may not be the same object due to the matching error problem. Moreover, two point clouds with small centroid distance or high degree of overlap do not necessarily belong to the same object. Therefore, it is necessary to consider the above three conditions together. The formula to determine whether two point clouds belong to the same object is as follows.

(22) $$P=w{\left(P_{ID},D,M\right)}^{T}$$

where w represents the weight vector. $P_{ID}$ represents whether two objects have the same ID, which equals 1 if they are the same and 0 if they are different. D represents the distance between the two centroids. M represents the degree of overlap of two point clouds. P represents the probability that two point clouds belong to the same object. The larger the P, the higher the probability that two point clouds belong to the same object. We choose a probability threshold K. When P>K, this indicates that the two point clouds belong to the same object. The centroids of the two matching point clouds are added to the calculation of pose estimation to optimize the camera pose.

## 4. Experiments and Analysis

All the experiments in this paper are completed under the Ubuntu20.04 system. The semantic information extraction part adopts Mask R-CNN and Deepsort based on the deep-learning framework of keras-2.1.5 and tensorflow_gpu-1.13.2, completed by python. The semantic mapping part adopts the ORB-SLAM2 system based on opencv-3.4.16, pcl-1.9.0, pangolin-0.6, eigen-3.3.8, vtk-7.1.1 and other dependency libraries, completed by C++. The computing power of the computer is 5.2. The experiments use cuda-10.0 and cudnn-7.4.1.5 for gpu acceleration, opencv for image data feature extraction and matching, pcl for mapping, point cloud segmentation, point cloud fusion, point cloud storage, pangolin for visual interface. Eigen provides a large number of linear algebras of matrices, matrices, vector operation functions, etc. G2o is a graph optimization solver, and the DBoW3 is used for camera relocalization and loop detection in the visual SLAM. The computer parameters of the experiments are shown in Table 1.

The semantic mapping algorithm in this paper is applicable to indoor office scenes. In all SLAM datasets, the TUM dataset is a classic indoor SLAM dataset, including the static scene, dynamic scene, object reconstruction scene and other scenes [66]. This paper conducts experiments and tests on six datasets in TUM using the semantic mapping algorithm, where, some images in the freiburg2_dishes datasets are used for labeling and training to generate the self-training weight, which is used to predict and generate the mask and label of the objects. Meanwhile, the other five datasets use the coco weight trained in advance to predict on the images. The task of our method is to optimize the camera pose, construct object-oriented semantic maps and reconstruct the multi-objective objects detected by the instance segmentation algorithm in the indoor scene.

### 4.1. Experiments on Self-Training Weight

The RGBD datasets of freiburg2_dishes in TUM are selected as the datasets of the experiments. The datasets contain 3007 color images and 3005 depth images. The main detection objects are three types of tableware in the image, including two bowls, one cup and one plate. The detection task is to complete the instance segmentation of four objects in the images, generate the detection box, semantic label and mask of each object, and obtain the pixel coordinates of the four objects according to the corresponding mask. In total, 750 images are selected from freiburg2_dishes for annotation to generate json files, which will be converted to voc format and put into Mask R-CNN for training. The specific parameters for training in Mask R-CNN are shown in Table 2.

In total, 750 images are selected for annotation and training in a dataset containing 3000 images. It is now common practice to divide the dataset into a training set, a validation set and a test set. Empirically, the training ratio is 0.8, the validation ratio is 0.1, and the test ratio is 0.1. In addition, the model terminates the experiment early if there is no decrease in validation loss for five consecutive epochs during training. The training set is used to train the parameters of the model, and the test set is used to evaluate the goodness of the model. The validation set is used to validate the model training effect in the training phase and is used to select the hyperparameters. Resnet101 is selected as the backbone of the model, and this network is used as the backbone in many instance segmentation networks due to its performance advantages, such as Mask R-CNN [36], Cascade Mask R-CNN [35], etc. The epoch and Bach size are set according to the performance of the computer. The training time for each epoch in the training is 2917 s. Overall, the parameters of our model are chosen empirically or with reference to the work of others and are not necessarily the best options. There is not a lot of difference in the overall performance and no major impact on subsequent tasks.

**Figure 3:**
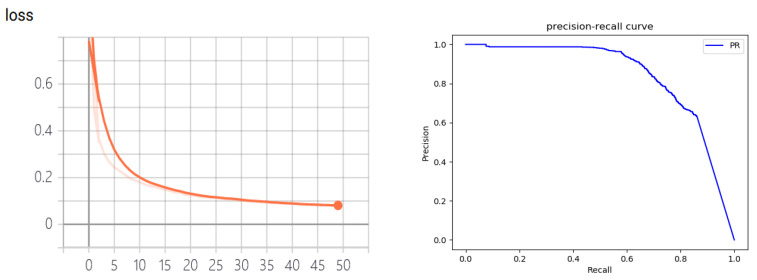
The training results of Mask R-CNN on freiburg2_dishes. The left image is the value loss diagram, and the right is the precision–recall diagram.

To ensure the accuracy of the pixel coordinates of the extracted objects, the detection performance of the Mask R-CNN needs to be verified. The mean average precision (mAP) is selected as the evaluation indicator to evaluate the detection performance of Mask R-CNN. The two important parameters of the instance segmentation network are precision and recall, and the calculation formula of the two parameters is

(23) $$precision=\frac{TP}{TP+FP}\;\;\;\;\;\;\;recall=\frac{TP}{TP+FN}$$

where TP (true positives) signifies that the object is divided into positive samples and is correct. TN (true negatives) signifies that the object is divided into negative samples and is correct. FP (false positives) signifies that the object is divided into positive samples and is incorrect. FN (false negatives) signifies that the object is divided into negative sample and is incorrect. The average precision (AP) in fact refers to the area under the precision and recall curve. The mAP refers to the mean value of the AP for all classes. The higher the mAP, the better the detection performance of the network. The training results of Mask R-CNN on freiburg2_dishes are shown in Figure 3.

**Figure 4:**
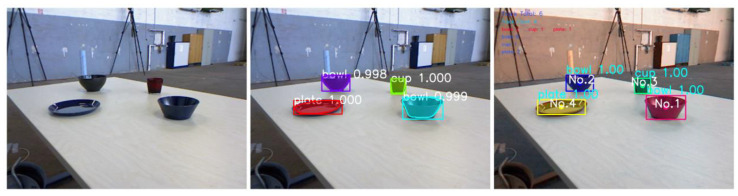
The detection results of freiburg2_dishes using self-training weight. From left to right, the first image is the original RGB image. The second is the detection result of Mask R-CNN. The third is the detection result of Mask R-CNN with Deepsort.

It can be seen that the value loss basically converges when the epoch approaches 50. The detection results of freiburg2_dishes using self-training weight are shown in Figure 4.

**Figure 5:**
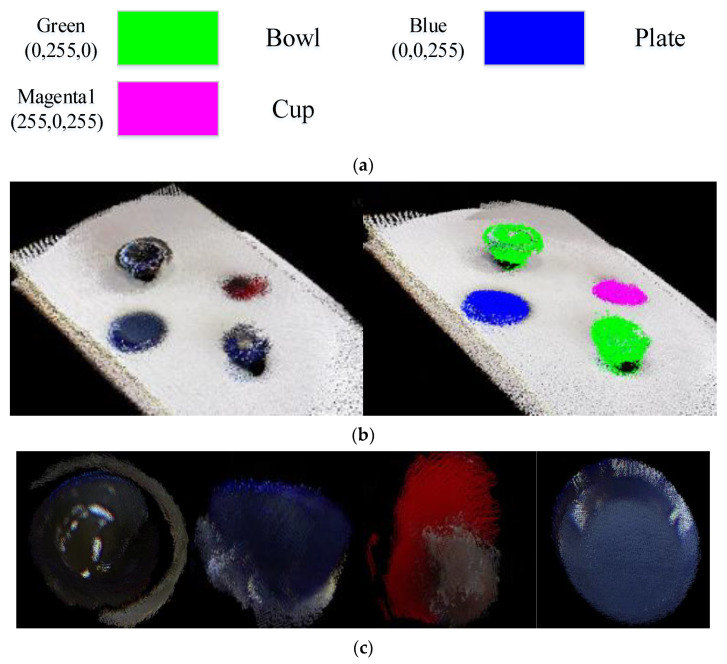
The results of semantic mapping of freiburg2_dishes using self-training weight. Figure (**a**) is the color information corresponding to three categories of objects in semantic map. Figure (**b**) is the dense point cloud map (**left**) and the dense semantic point cloud map (**right**). Figure (**c**) is the dense point cloud model of each object of freiburg2_dishes (bowl, bowl, cup, plate).

As shown in Figure 4, each object on the image has its own ID after the target tracking algorithm is added to Mask R-CNN. After instance segmentation and target tracking, the RGB images, depth images and the txt files with the pixel coordinates of each object on each key frame are input to the ORB-SLAM2 system to obtain the semantic map of the environment and the dense point cloud model of each object. The results of dense mapping and dense semantic mapping on freiburg2_dishes using self-training weight are shown in Figure 5.

As shown in Figure 5, the algorithm works well for objects with obvious features, such as a bowl and a plate, but not for objects with no obvious features, such as a cup. The reason is that the measured depth of the edge of the object is usually inaccurate, and the positioning of orbslam2 also has errors, and the mask obtained by Mask R-CNN is not perfect, which causes the point cloud of each object to be not fine enough.

### 4.2. Experiments on Coco Weight

**Figure 6:**
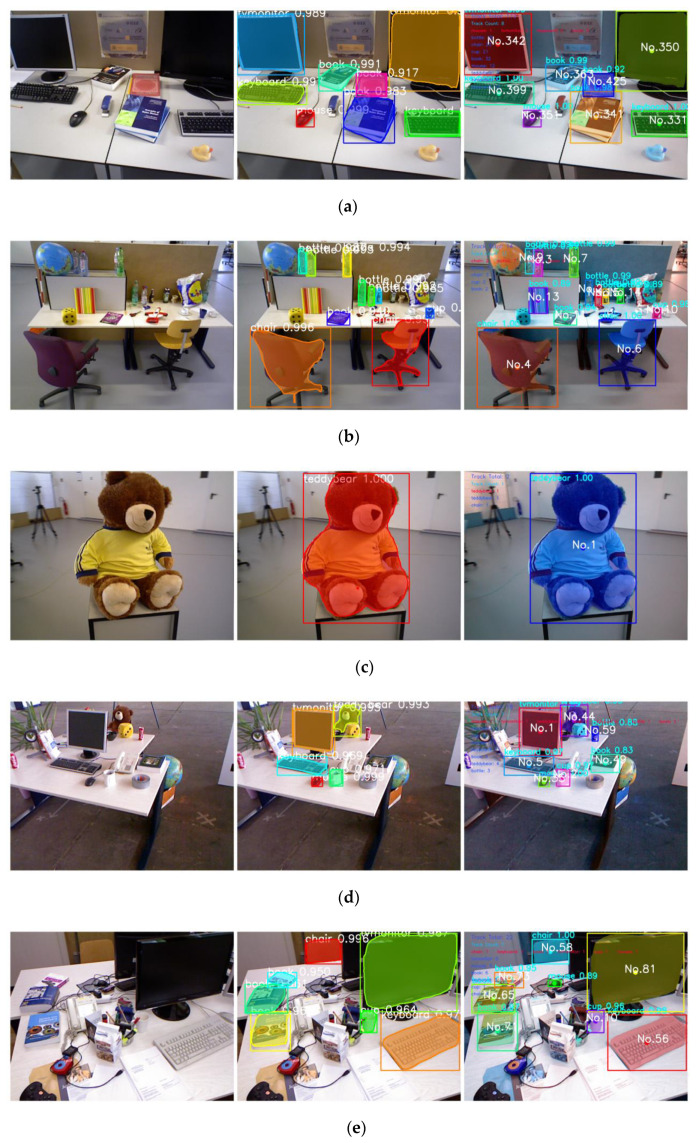

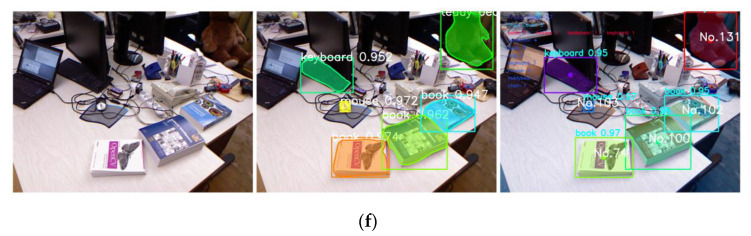
The results of target tracking and instance segmentation on TUM datasets using coco weight. From left to right, the first figure is the original RGB image. The second is the detection result of Mask R-CNN. The third is the detection result of Mask R-CNN with Deepsort. Figure (**a**–**f**) are the detection results of the back of freiburg3_office, the front of freiburg3_office, freiburg3_teddy, freiburg2_desk, freiburg1_desk and freiburg1_room respectively.

The experiments are conducted on five RGBD datasets in TUM using the coco weight, including freiburg1_desk, freiburg1_room, freiburg2_desk, freiburg3_office, freiburg3_teddy. The mask_rcnn_coco.h5, which was already trained on the coco datasets, is selected as the weight used for detection. Eight categories of objects in the coco datasets are selected for detection in our method, including chair, cup, TV monitor, teddy bear, bottle, book, keyboard and mouse. The detection task is to complete the instance segmentation in each image and target tracking of the objects in an image sequence and record the pixel coordinates of each object in the corresponding txt files. The results of target tracking and instance segmentation on TUM datasets using the coco weight are shown in Figure 6.

As shown in Figure 6, the detection result of Mask R-CNN includes the semantic label, score, detection box and mask. The detection result of Mask R-CNN with Deepsort includes the semantic label, score, detection box, mask and tracked ID. Multiple objects in the image are tracked to establish the data association between different images. It can be seen from (a) and (b) that only using Mask R-CNN for detection may result in partial missed detection, such as the book with ID 13 in (a) and the book with ID 49, the cup with ID 59 in (d). Mask R-CNN with Deepsort can predict the state of the system in the next frame according to the detection results of Mask R-CNN and the motion model of the system. Therefore, there will be less missed detection in the converged system, which will make the point cloud model of the object more complete in the subsequent point cloud mapping. Moreover, instance segmentation will produce some wrong results. For example, windows are detected as TV monitors, cups are detected as mice, which eventually leads to some errors in semantic mapping. The target tracking algorithm can reduce wrong results through data associations.

**Figure 7:**
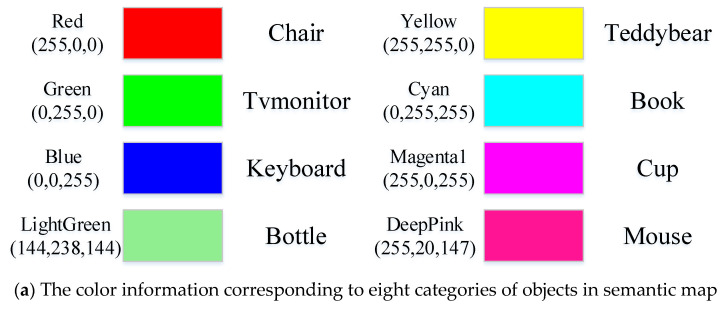

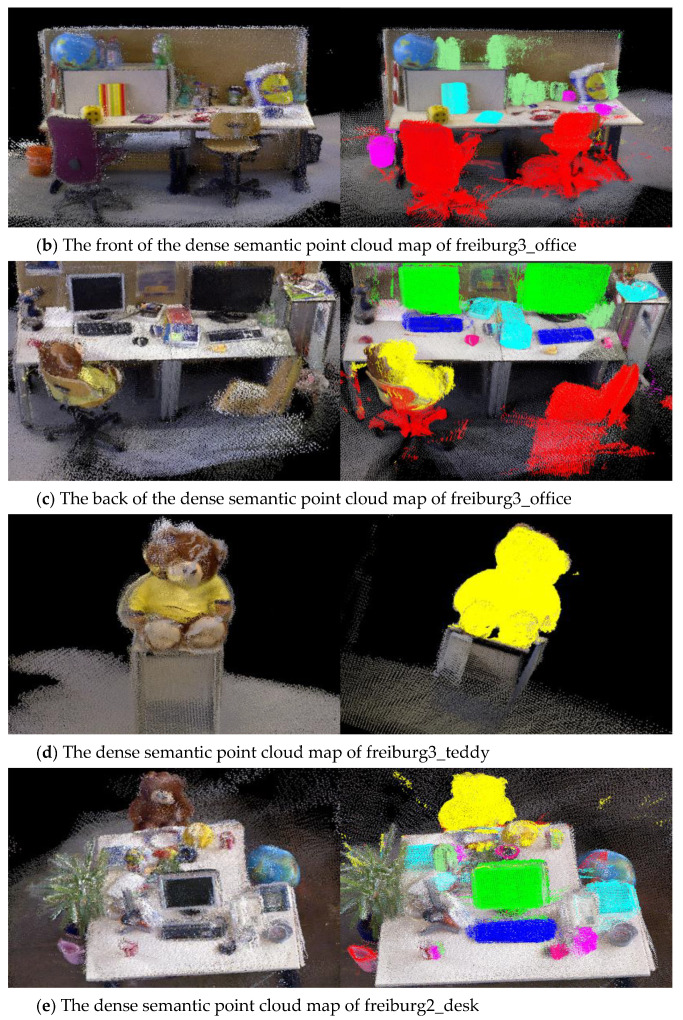

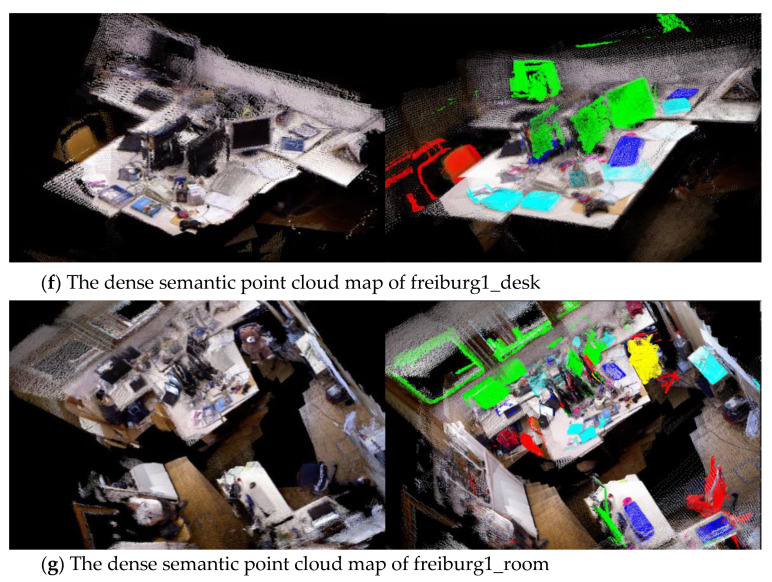
The results of semantic mapping of five datasets in TUM using coco weight. Figure (**a**) shows the color corresponding to the objects. Figures (**b**–**g**) show the dense point cloud map (left) and the dense semantic point cloud map (right) of the 5 datasets.

The above semantic information extracted by instance segmentation and target tracking are input to the ORB-SLAM2 system along with the RGB images and depth images to obtain the dense point cloud map, the dense semantic point cloud map of the environment and the dense point cloud model of each object. The results of dense point cloud mapping and dense semantic point cloud mapping of the five datasets in TUM using the coco weight are shown in Figure 7.

**Figure 8:**
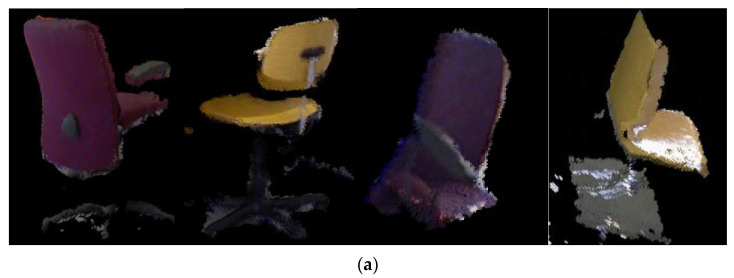

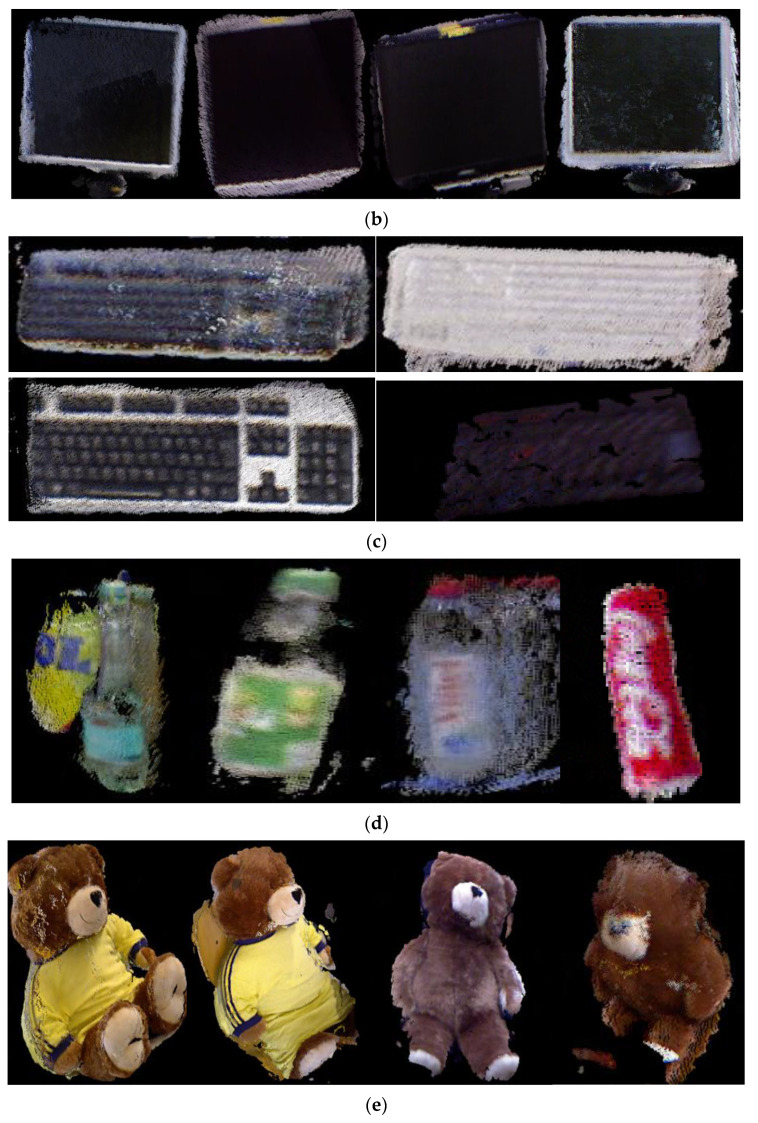

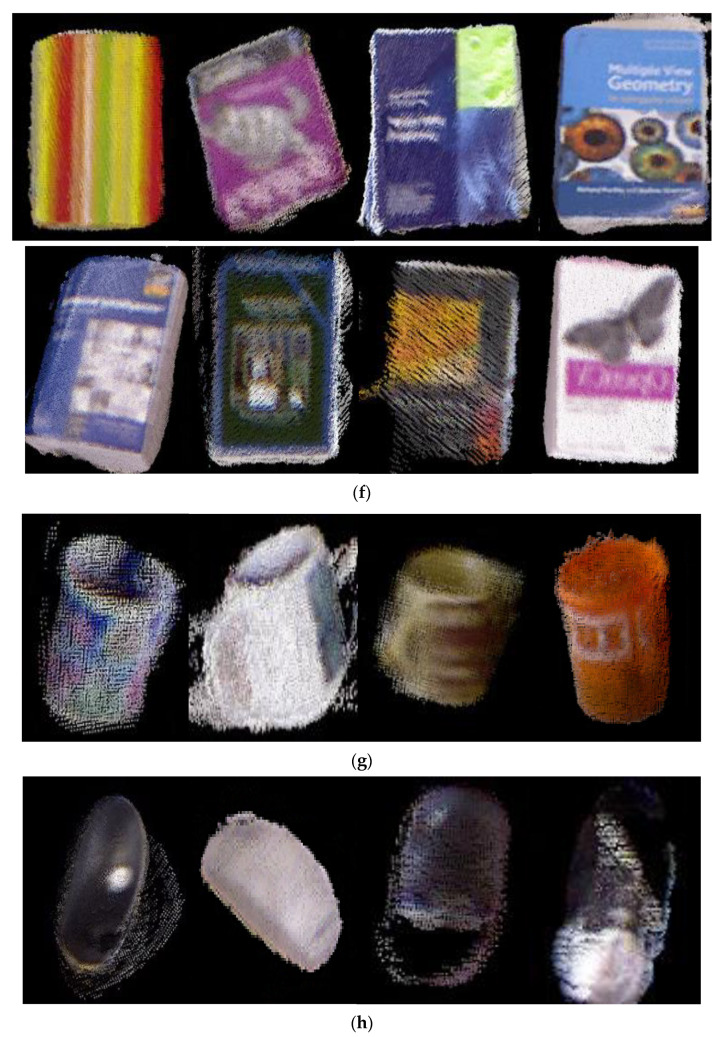
The dense point cloud model of the eight categories of objects. Figure (**a**–**h**) are the point clouds of the chair, TV monitor, keyboard, bottle, teddy bear, book, cup, mouse respectively.

As shown in Figure 7, the dense point cloud map is added as a comparison to make the results of semantic mapping look clearer. There is a lot of noise in the boundary of the object when it is converted to 3D point cloud due to the imperfect boundary segmentation of the object in semantic segmentation, which can be mitigated by adjusting the parameters of the outpoint filtering algorithm. It can be seen that the semantic maps do not change in the overall scene but add colors to each target object to distinguish and facilitate scene understanding. The object to be reconstructed is the object marked with a color. The dense point cloud model of the eight categories of objects obtained by matching and fusion between point clouds is shown in Figure 8.

As shown in Figure 7 and Figure 8, the algorithm works well for objects with obvious features, such as a TV monitor, book, teddy bear and keyboard, but not for objects with no obvious features, such as a cup, bottle, mouse and chair. This is partly because the depth data of the object edge in the depth map collected by the RGB-D camera are inaccurate. Another reason is that there are errors in the camera pose estimated by ORB-SLAM2, which leads to ghosting of the map after fusion, such as the bottle, keyboard and cup shown in Figure 8c,d,g. In addition, the mask obtained by Mask R-CNN is not perfect. In the process of mapping, only when the camera rotates 360 degrees around the object can we obtain a complete point cloud model of the object. Otherwise, the point cloud model will be missing somewhere. For example, when we view the point cloud model from another angle, it may be missing a piece. Adding a target tracking algorithm to the process of extracting semantic information can provide a priori information for matching and fusing objects in a 3D space, thus optimizing the camera poses and constructing globally consistent point clouds of objects.

The centroid of the dense point cloud can be calculated after obtaining the point cloud model of each object. There are two ways to evaluate the accuracy and effectiveness of the algorithm. The first method is to compare the number of detected objects (the number of final point cloud models, expressed by D) with the number of actual objects (the ground truth value, expressed by GT) in the five datasets, and the results are shown in Table 3 (only the above eight categories of objects are calculated).

The difference between D and GT can be used to express the accuracy of the algorithm, and the formula of the difference can be expressed as

(24) $$\mathrm{error}=\frac{\mathrm{abs}(D-GT)}{GT}\times 100\%$$

The smaller the error, the more accurate the algorithm. It is worth mentioning that D is always greater than GT because the IDs of objects always switch in target tracking, despite the point cloud models with the same label and close distance being merged. As shown in Table 3, the number of detected objects (D) is greater than or equal to the number of actual objects in all datasets. This is because an object may correspond to multiple tracking IDs, or some detection areas that are not target objects are detected as objects. The more objects in the environment, the greater the error of the algorithm. It can be seen from the results of the error that our algorithm can segment the point cloud model of objects in the environment with a high accuracy.

The second method is to compare the objects’ centroids computed by the mapping algorithm with the ground truth of the objects’ centroids. The TUM datasets do not provide the true position of each object but the ground truth pose, which is measured by an external (very advanced) motion capture device. The ground truth pose can be regarded as a standard answer to calculate the ground truth of the objects’ centroids in a 3D space. The estimated camera pose takes the camera pose of the first frame as the origin of the world coordinate system, and the ground truth camera pose takes a fixed point in space as the origin of the world coordinate system. Therefore, the ground truth camera pose needs to be converted to the estimated world coordinate system. The objects’ centroids calculated by the estimated pose and the ground truth pose are shown in Table 4 (take fr3_office, for example).

The error represents the distance of the estimated centroid and the ground truth centroid. The smaller the error, the higher the accuracy of the algorithm. It can be seen from Table 4 that the average error of the position of the object calculated for the eight categories of objects in the fr3_office dataset is 0.0109 m, which means that the positioning algorithm has a high accuracy. It can be seen from the table that the larger the volume of the object, the worse the positioning accuracy, which is consistent with our common sense. Moreover, the higher the positioning accuracy of the algorithm, the more accurate the positioning of the object. Fortunately, the positioning accuracy of orbslam2 is very high, and the absolute pose error is 0.0094 m in the fr3_office dataset. In order to further verify the effectiveness of the algorithm, the average positioning error of each dataset is calculated and is shown in Table 5.

The average positioning error of objects refers to the distance of the objects’ three-dimensional centroids calculated by using the estimated and true values of the camera pose. The maximal average positioning error (m) of objects is 0.347 m and the minimal is 0.00375 m. Why can some objects have such large errors? The reason is that the observation angles of each object in each picture are different. Similarly, the pose errors of each different key frame are different, and each object with different angles will generate a point cloud. Finally, when the point cloud is fused, some redundancy may occur, resulting in a certain degree of offset of the object’s centroid. However, it can be seen in general that, from the above data, the algorithm has a high accuracy for small objects and a poor accuracy for large objects. Overall, the system has a high positioning accuracy.

If collision and path planning are not considered, other map information in the environment is redundant, except for the map information of objects. The number of point clouds of each dataset is shown in Table 6. The number of point clouds of each dataset is reduced by 45.3%, 59.8%, 57.6%, 61.5% and 54.3%, respectively, after segmenting the objects from the environment, which effectively reduces the system memory usage and removes the redundant information from the environment.

### 4.3. Comparison with Existing Methods

Quantitative comparison. In the study of SLAM systems, the absolute trajectory error (ATE) or relative pose error (RPE) are generally used to evaluate the merits of a SLAM system, and both evaluation criteria revolve around the pose estimation results of SLAM systems. In this paper, the absolute positional error is used to evaluate the performance of SLAM systems. The following Table 7 shows the comparison of our method with the existing methods.

As can be seen from the above table, our method shows better performance in most of the datasets. Compared with the ORB-SLAM2 system, our approach has an overall performance improvement with the addition of semantic constraints. It is worth noting that our system is not yet up to the real-time requirement, which requires further tight integration of semantics and SLAM to further improve the performance of the system.

Functional comparison. There are no recognized reliable evaluation criteria for map construction effects. Some classical semantic slam systems are selected for comparison with the proposed approach in this paper, mainly around the implemented functions. The comparison results are shown in Table 8.

Most of the current semantic SLAM systems focus on semantic mapping and semantic-assisted localization while ignoring the individual entities in the scene. The method proposed in this paper constructs an object-oriented semantic map of the scene while completing the reconstruction and localization of multi-target objects and optimizing the camera pose with semantic constraints, which has certain implications for the subsequent development of semantic SLAM.

Specifically, a beautiful object-based semantic map is constructed by using prior information in SLAM++, but the 3D model of the object needs to be known in advance, and the real scene cannot be restored [55]. CNN-SLAM combines the depth map predicted by CNN with the depth map estimated by monocular SLAM to obtain a more accurate depth map and uses this to build a scenario-oriented semantic map, but the system is more concerned with depth estimation than with semantic maps [54]. Sunderhauf combines SSD with ORB-SLAM2 to construct object-based semantic maps, but the experiment only includes four objects, and the mapping results perform well only for square objects, such as monitors, books and keyboards [58]. SemanticFusion can construct a dense surfel reconstruction from a video sequence but not to the level of a single object [50]. Mask-Fusion combines Mask R-CNN with Elastic-fusion to construct a real-time, object-aware, semantic and dynamic RGB-D SLAM system, which can reconstruct each object in the environment [63]. However, two GPUs with very good performance are required to accelerate in the actual running process in Mask-Fusion, and even so, the running speed is still very slow, which is difficult to meet the real-time performance requirement, and the map effect is poor. In our system, the process of extracting semantic information is the process of pre-processing, and the main running tasks are optimizing the camera pose, constructing semantic maps and reconstructing multi-target objects. In fact, eight categories of objects are used for detection and semantic mapping using coco weights in the experiments, and most objects in the actual scene can be reconstructed. In addition, our system can construct the dense semantic point cloud map and output point cloud model of each object to reconstruct each object with a high accuracy and small positioning error.

Overall, the method proposed in the paper has clear advantages. First, with the introduction of semantic information, the camera poses of the traditional SLAM system are optimized, which indicates that the robot is positioned more accurately in the scene. Second, the introduced semantic information can convert the traditional point cloud map into an object-oriented point cloud map, which allows the robot to interact with individuals in the environment. For example, tasks such as asking the robot to pick up a book on a table can be achieved because the map clearly shows the location of each object, and the system has sufficient localization accuracy, except for individual errors. Again, our system is able to output point cloud maps of each object in the environment, and these point cloud maps can be used for subsequent 3D reconstruction tasks, such as using TSDF-based surface reconstruction, which can result in beautiful object models. Of course, our proposed method also has some disadvantages. First, the system is currently not real time because it contains a pre-processing step for instance segmentation, and the mask R-CNN is slow in detection, although it has a high accuracy. In order to make the system run in real time, both detection accuracy and detection speed must be considered. Second, when optimizing the camera pose, we only track and match the center of the mass of the target point cloud without using the full semantic information. If the full semantic information could be used, the camera pose could be further optimized.

## 5. Conclusions

Inspired by image processing methods, such as deep-learning, target detection and instance segmentation algorithms, we propose a method for constructing object-oriented semantic maps that maintains individual objects as the key entities in the map, which builds on SLAM, instance segmentation and target tracking. Experiments show that the 3D semantic point cloud map, which only contains objects, reduces the number of points by about 50% compared with the dense point cloud map of the complete environment. The average positioning error of the eight categories of objects in TUM datasets is very small, which means that the positioning algorithm has a high accuracy. In the tests on the five TUM datasets, the absolute positional error of the camera is also reduced with the introduction of semantic constraints, and the positioning performance of the system is improved. At the same time, our algorithm can segment the point cloud model of objects in the environment with a high accuracy, which is of great practical importance in practical engineering applications.

Unfortunately, there are some shortcomings in the methodology of this paper, which need to be improved. Our method uses semantic information to improve the accuracy of SLAM localization and complete semantic map building but does not currently meet the real-time requirements, nor does it use data association to improve the accuracy of semantic information extraction. Our future work will focus on the above two issues.

## Figures and Tables

**Table 1:** The computer parameters of the experiments.

Name	Model	Remarks
Operating System	Ubuntu 20.04	/
Graphic Processing Unit (GPU)	NVIDIA Quadro M2000	4G
Central Processing Unit (CPU)	Intel^®^ Xeon(R) CPU E5-1620 v4	3.5 GHz * 8
Random Access Memory (RAM)	DDR4	32G
Hard Disk	SSD256G + HDD 1000G	Samsung

**Table 2:** The specific parameters for training in Mask R-CNN.

Parameters	Quantity
backbone	Resnet101
Epoch	50
Number of pictures	750
Training ratio	0.8
Validation ratio	0.1
Test ratio	0.1
Bach size	1
Training time(s/epoch)	2917
class_names	bowl, plate, cup

**Table 3:** The number of detected objects and actual objects.

Datasets	The Number of Detected Objects (D)	The Number of Actual Objects (GT)	Error
fr3_office	36	30	20.0%
fr3_teddy	1	1	0.0%
fr2_desk	18	16	12.5%
fr1_desk	16	15	6.7%
fr1_room	21	20	5.0%

**Table 4:** The objects’ centroids calculated by the estimated pose and the ground truth.

Objects	Tracked ID	Method	Centroids	Error (m)
chair	6	estimated	[0.585, 0.343, 2.32]	0.0237
		ground truth	[0.591, 0.352, 2.34]	
TV monitor	389	estimated	[0.592, −0.713, 3]	0.00823
		ground truth	[0.593, −0.705, 3]	
keyboard	513	estimated	[0.565, −0.614, 3.24]	0.0116
		ground truth	[0.576, −0.612, 3.24]	
bottle	3	estimated	[−0.513, −0.78, 2.4]	0.00828
		ground truth	[−0.511, −0.773, 2.4]	
teddy bear	188	estimated	[0.778, −0.966, 3.81]	0.0131
		ground truth	[0.776, −0.953, 3.81]	
book	11	estimated	[−0.203, −0.0962, 2.28]	0.00997
		ground truth	[−0.198, −0.0899, 2.29]	
cup	41	estimated	[−1.37, 0.143, 2.92]	0.0098
		ground truth	[−1.37, 0.147, 2.93]	
mouse	402	estimated	[0.252, −0.701, 3.41]	0.00312
		ground truth	[0.254, −0.7, 3.4]	

**Table 5:** The average positioning error (m) of objects in five datasets.

Objects	fr3_Office	fr3_Teddy	fr2_Desk	fr1_Room	fr1_Desk
chair	0.0235	/	0.146	0.266	0.232
TV monitor	0.00927	/	0.266	**0.347**	0.0839
keyboard	0.0118	/	0.0908	0.347	0.123
bottle	0.0154	/	0.0557	0.0347	0.00387
teddy bear	0.0177	0.00186	0.133	0.189	/
book	0.00972	/	0.0238	0.161	0.00946
cup	0.0167	/	0.0199	0.0539	0.0366
mouse	0.00656	/	**0.00375**	0.0979	0.0846

**Table 6:** The number of point clouds of five datasets.

Datasets	Number of Original Point Clouds	Number of Point Clouds with Objects Only	Reduction
fr3_office	4,814,372	2,635,597	45.3%
fr3_teddy	8,451,663	3,395,009	59.8%
fr2_desk	3,090,597	1,195,625	57.6%
fr1_room	2,581,204	993,299	61.5%
fr1_desk	364,226	166,546	54.3%

**Table 7:** Comparison of the localization performance of our method with existing SLAM systems. RMSE (m).

Sequence	ORB-SLAM2 [41]	Elastic-Fusion [43]	DVO-SLAM [38]	Fusion++ [40]	Ours
fr3_office	0.0121	0.017	0.035	0.1082	**0.0115**
fr3_teddy	**0.0225**	0.048	0.046	0.1535	0.0259
fr2_desk	0.0095	0.071	0.017	0.1144	**0.0086**
fr1_room	0.0474	0.068	**0.043**	0.2356	0.0452
fr1_desk	0.0163	0.020	0.021	0.0499	**0.0153**

**Table 8:** Comparison of the function of our method with existing semantic SLAM systems.

Semantic SLAM System	Semantics	Scenario-Oriented Semantic Maps	Object-Oriented Semantic Maps	Semantic Help SLAM Positioning	Objects Location	Objects Reconstruction
SLAM++ [55]	√		√			
Meaningful maps [58]	√		√			
CNN-SLAM [54]	√	√				
SemanticFusion [50]	√	√		√		
MaskFusion [63]	√		√	√		√
Ours	√		√	√	√	√

## Data Availability

The data that support the findings of this study are available from the corresponding author upon reasonable request.
